# Prediagnostic circulating concentrations of plasma insulin‐like growth factor‐I and risk of lymphoma in the European Prospective Investigation into Cancer and Nutrition

**DOI:** 10.1002/ijc.30528

**Published:** 2016-12-27

**Authors:** Aurora Perez‐Cornago, Paul N. Appleby, Sarah Tipper, Timothy J. Key, Naomi E. Allen, Alexandra Nieters, Roel Vermeulen, Sandrine Roulland, Delphine Casabonne, Rudolf Kaaks, Renee T. Fortner, Heiner Boeing, Antonia Trichopoulou, Carlo La Vecchia, Eleni Klinaki, Louise Hansen, Anne Tjønneland, Fabrice Bonnet, Guy Fagherazzi, Marie‐Christine Boutron‐Ruault, Valeria Pala, Giovanna Masala, Carlotta Sacerdote, Petra H. Peeters, H. B(as) Bueno‐de‐Mesquita, Elisabete Weiderpass, Miren Dorronsoro, J. Ramón Quirós, Aurelio Barricarte, Diana Gavrila, Antonio Agudo, Signe Borgquist, Ann H. Rosendahl, Beatrice Melin, Nick Wareham, Kay‐Tee Khaw, Marc Gunter, Elio Riboli, Paolo Vineis, Ruth C. Travis

**Affiliations:** ^1^ Nuffield Department of Population Health, Cancer Epidemiology UnitUniversity of OxfordOxfordUnited Kingdom; ^2^ Clinical Trial Service Unit and Epidemiological Studies Unit, University of OxfordOxfordUnited Kingdom; ^3^ Center for Chronic Immunodeficiency, Molecular EpidemiologyUniversity Medical Center FreiburgFreiburgGermany; ^4^ Institute for Risk Assessment Sciences, Division Environmental Epidemiology, Utrecht UniversityUtrechtThe Netherlands; ^5^ Centre d'Immunologie de Marseille‐Luminy, Université d'Aix‐Marseille UM2, Inserm, U1104, CNRSMarseilleFrance; ^6^ Unit of Infections and Cancer (UNIC), IDIBELL, Institut Català d'Oncologia, 08907 L'Hospitalet de LlobregatBarcelonaSpain; ^7^ CIBER Epidemiología y Salud Pública (CIBERESP)MadridSpain; ^8^ Division of Cancer EpidemiologyGerman Cancer Research Center (DKFZ)HeidelbergGermany; ^9^ Department of EpidemiologyGerman Institute of Human Nutrition Potsdam‐RehbrückeNuthetalGermany; ^10^ Hellenic Health FoundationAthensGreece; ^11^ Department of Hygiene, Epidemiology and Medical Statistics, WHO Collaborating Center for Nutrition and Health, Unit of Nutritional Epidemiology and Nutrition in Public HealthUniversity of Athens Medical SchoolGreece; ^12^ Department of Clinical Sciences and Community Health Università degli Studi di MilanoItaly; ^13^ Danish Cancer Society Research CenterCopenhagenDenmark; ^14^ Université Paris‐Saclay, Université Paris‐Sud, UVSQ, CESP, INSERMVillejuifFrance; ^15^ Gustave RoussyVillejuifFrance; ^16^ CHU Rennes, University Rennes 1RennesFrance; ^17^ Epidemiology and Prevention Unit, Fondazione IRCCS Istituto Nazionale dei TumoriMilanoItaly; ^18^ Molecular and Nutritional Epidemiology Unit, Cancer Research and Prevention Institute—ISPOFlorenceItaly; ^19^ Unit of Cancer Epidemiology, AO Citta' della Salute e della Scienza‐University of Turin and Center for Cancer Prevention (CPO‐Piemonte)TurinItaly; ^20^ Department of Epidemiology, Julius Center for Health Sciences and Primary CareUniversity Medical Center UtrechtThe Netherlands; ^21^ Department of Epidemiology and Biostatistics, MRC‐PHE Centre for Environment and Health, School of Public HealthImperial CollegeLondonUnited Kingdom; ^22^ Department for Determinants of Chronic Diseases (DCD)National Institute for Public Health and the Environment (RIVM)BilthovenThe Netherlands; ^23^ Department of Epidemiology and Biostatistics, School of Public HealthImperial College LondonLondonUnited Kingdom; ^24^ Department of Social & Preventive Medicine, Faculty of MedicineUniversity of MalayaKuala LumpurMalaysia; ^25^ Department of Community Medicine, Faculty of Health SciencesUniversity of Tromsø, The Arctic University of NorwayTromsøNorway; ^26^ Department of Research, Cancer Registry of NorwayInstitute of Population‐Based Cancer ResearchOsloNorway; ^27^ Department of Medical Epidemiology and BiostatisticsKarolinska InstitutetStockholmSweden; ^28^ Genetic Epidemiology Group, Folkhälsan Research CenterHelsinkiFinland; ^29^ Basque Regional Health Department San SebastianPublic Health Direction and Biodonostia‐ CiberespSpain; ^30^ Public Health DirectorateAsturiasSpain; ^31^ Navarra Public Health InstitutePamplonaSpain; ^32^ Navarra Institute for Health Research (IdiSNA)PamplonaSpain; ^33^ Department of EpidemiologyMurcia Regional Health Council, IMIB‐ArrixacaMurciaSpain; ^34^ Unit of Nutrition and Cancer, Cancer Epidemiology Research ProgramCatalan Institute of Oncology‐IDIBELL, L'Hospitalet de LlobregatBarcelonaSpain; ^35^ Department of Clinical Sciences Lund, Division of Oncology and PathologyLund University, Faculty of MedicineLundSweden; ^36^ Department of Radiation SciencesOncology Umeå UniversityUmeåSweden; ^37^ MRC Epidemiology Unit, University of CambridgeCambridgeUnited Kingdom; ^38^ University of Cambridge School of Clinical MedicineCambridgeUnited Kingdom; ^39^ Section of Nutrition and Metabolism, International Agency for Research on CancerLyonFrance

**Keywords:** prospective, lymphoma, non‐Hodgkin lymphoma, plasma, IGF‐I, EPIC cohort, nested case–control

## Abstract

Insulin‐like growth factor (IGF)‐I has cancer promoting activities. However, the hypothesis that circulating IGF‐I concentration is related to risk of lymphoma overall or its subtypes has not been examined prospectively. IGF‐I concentration was measured in pre‐diagnostic plasma samples from a nested case–control study of 1,072 cases of lymphoid malignancies and 1,072 individually matched controls from the European Prospective Investigation into Cancer and Nutrition. Odds ratios (ORs) and confidence intervals (CIs) for lymphoma were calculated using conditional logistic regression. IGF‐I concentration was not associated with overall lymphoma risk (multivariable‐adjusted OR for highest versus lowest third = 0.77 [95% CI = 0.57–1.03], *p*_trend_ = 0.06). There was no statistical evidence of heterogeneity in this association with IGF‐I by sex, age at blood collection, time between blood collection and diagnosis, age at diagnosis, or body mass index (*p*_heterogeneity for all_ ≥ 0.05). There were no associations between IGF‐I concentration and risk for specific BCL subtypes, T‐cell lymphoma or Hodgkin lymphoma, although number of cases were small. In this European population, IGF‐I concentration was not associated with risk of overall lymphoma. This study provides the first prospective evidence on circulating IGF‐I concentrations and risk of lymphoma. Further prospective data are required to examine associations of IGF‐I concentrations with lymphoma subtypes.

## Abstract 2

**What's new?**

Insulin‐like growth factor I does not appear to influence lymphoma risk, according to new results. IGF‐I can promote some cancers, but there hasn't been a prospective epidemiological study examining the link between IGF‐I concentration and lymphoma risk. To uncover a link, these authors arranged a NESTED case–control study with participants from the European Prospective Investigation into Cancer and Nutrition (EPIC). They tested for IGF‐I in pre‐diagnosis samples and found no association between the factor and overall lymphoma risk, nor with any subtype, although the number of cases was small for each subtype, and further studies are necessary.

**Abbreviations**
B‐CLL: B‐cell chronic lymphocytic leukemia; BCL: B‐cell lymphoma; BMI: body mass index
DLBCL: diffuse large B‐cell lymphoma
EPIC: European Prospective Investigation into Cancer and Nutrition
FL: follicular lymphoma
HL: Hodgkin lymphoma
IDS: Immunodiagnostic Systems
IGFs: insulin‐like growth factors
IARC: International Agency for Research on Cancer
MM: multiple myeloma
NHL: non‐Hodgkin lymphoma
ORs: odds ratios
UK: United Kingdom
WHO: World Health Organization

Despite the major impact of lymphoma on public health, relatively little is known about the aetiology of this group of malignancies, and the few established risk factors include age, family history, genetic factors, immunosuppression and viral infections.^1^ Insulin‐like growth factors (IGFs) have potential cancer promoting activities and circulating concentrations of IGF‐I have been shown to be associated with risk for developing several types of cancer.^2^, ^3^ However, the hypothesis that IGF‐I is related to overall lymphoma risk has not yet been examined prospectively.

IGF‐I is a polypeptide growth factor that is primarily produced in the liver under the stimulus of growth hormone.^4^ It is involved in the promotion of cell growth and prevention of apoptosis in many tissue types, including cancer cells in several malignancies, and can act in an endocrine, paracrine or autocrine manner.^4^ Evidence from *in vitro* studies and clinical reports supports a potential role of IGFs in the aetiology of hematological tumours, including lymphoma.^5^ Elevated IGF‐I concentrations may activate the PI3‐K/AKT and β1‐integrin signalling pathways in lymphoma cells, contributing to carcinogenesis,^6^ and there is some experimental evidence that IGF‐I may induce growth and survival of lymphoma cells.^7^

Lymphomas include a range of heterogeneous malignancies that originate from lymphocytes and recent evidence suggests aetiological heterogeneity of different lymphoma subtypes. IGF‐I has been found to be mitogenic for multiple myeloma (MM) cells both *in vitro* and *in vivo*.^7^, ^8^ However, there are few published data on circulating concentrations of IGFs in patients with haematological malignancies 9, 10, 11 and there have been no prospective investigations of IGF‐I and overall lymphoma incidence.

In this study, we will examine the relationship between pre‐diagnostic circulating concentration of IGF‐I and subsequent risk of overall lymphoma and lymphoma subtypes in the European Prospective Investigation into Cancer and Nutrition (EPIC).

## Material and Methods

### Study cohort

The EPIC cohort consists of approximately 500,000 individuals recruited between 1992 and 2000 from 23 centres in 10 European countries (Denmark, France, Germany, Greece, Italy, The Netherlands, Norway, Spain, Sweden, and the United Kingdom). The main aim of this cohort was to investigate the relationship between nutrition and cancer. Participant eligibility within each cohort was based essentially on geographic or administrative boundaries, mostly aged 35 to 70 years.^12^ Participants were mostly recruited from the general population, but there were some exceptions. Participants residing in Spain and Italy (Ragusa and Turin) comprise blood donors and their spouses, members of several health insurance schemes, employees of several enterprises, civil servants and the general population. Also, the Oxford cohort (UK) included a large number of vegetarians and health‐conscious participants. Participants provided detailed information on dietary and non‐dietary factors at recruitment, which took place between 1992 and 2000, and about 400,000 individuals also provided a blood sample. Follow‐up aimed at identifying cancer cases was mainly by population‐based cancer registries. In France, Germany and Greece, follow‐up was based on a combination of methods, including health insurance records, cancer and pathology registries, as well as active follow‐up through participants or relatives; self‐reported incident cancers were verified through medical records. All participants gave informed consent, and approval of the study was obtained from the Internal Review Board of the International Agency for Research on Cancer (IARC) (Lyon, France) and from ethics committees at the participating institutions. Further details of the methods of recruitment and study design have been described in detail elsewhere.^12^

Whole blood was aliquoted generally within 24 hr into separate plasma, serum, buffy coat and erythrocyte fractions and stored in liquid nitrogen (−196°C) at IARC (except for Denmark and Sweden where all samples were stored locally).

### Selection of cases and controls

The present nested case–control study includes lymphoma cases diagnosed after blood collection and individually matched control participants from nine participating countries: Denmark, Germany, Greece, Italy, The Netherlands, Norway, Spain, Sweden and the UK. Due to incomplete coding for lymphoid neoplasms, participants from France were not included in the current analysis (*n* = 68,050). In Germany and Greece, follow‐up is active and is achieved through checks of health insurance records, cancer and pathology registries or *via* self‐reported questionnaires, while in the remaining countries it is achieved through linkage to national cancer registries.

Participants were eligible for this analysis if they had information available on the date of blood collection and did not have a history of cancer (except non‐melanoma skin cancer) at the time of the blood collection (*n* = 27,089 were excluded). Cases were eligible for inclusion if they were diagnosed with lymphoma after the date of blood collection until the end of the follow‐up period. Each case patient was matched to one control participant, selected at random among appropriate risk sets consisting of all cohort members alive and free of cancer (except non‐melanoma skin cancer) after the same amount of follow‐up time as the index case. An incidence density sampling protocol for control selection was used, such that controls could include participants who became a case later in time, while each control participant could also be sampled more than once. Matching criteria were: recruitment centre, sex, age at recruitment (±12 months), date at recruitment (±3 months), duration of follow‐up, and time of day (±1 hr) and fasting status at blood collection (Supporting Information Figure 1). The final sample comprised 1,072 cases and 1,072 controls with a mean follow‐up time of 9 years (SD: 2 years).

While lymphomas have traditionally been classified as either Hodgkin lymphoma (HL) or non‐Hodgkin lymphoma (NHL), in this analysis lymphomas were categorised, as in previous EPIC lymphoma papers,^13^ according to the current World Health Organization (WHO) classification of haematopoietic and lymphoid tumours,^14^ which differentiates between B‐cell neoplasms, T‐cell tumours, HL, and based on distinct morphologic, immunophenotypic, and genetic features. Cases comprised 897 B‐cell lymphoma (BCL), 34 T‐cell, 51 HL, and 90 other subtypes of lymphoma. The 897 BCL cases were further categorised into 124 diffuse large B‐cell lymphoma (DLBCL), 115 follicular lymphoma (FL), 184 B‐cell chronic lymphocytic leukemia (B‐CLL), 237 MM 237 other subtypes of BCL [*i.e*. those cases for which the BCL subtype is unknown or does not fall within the more common BCL subtypes (*i.e*. DBCL, FL, B‐CLL or MM)].

### Laboratory assays

Plasma IGF‐I concentrations were measured using the automated IDS‐iSYS immunoassay system from Immunodiagnostic Systems (IDS) Ltd. at the Cancer Epidemiology Unit laboratory (University of Oxford, UK). Two quality control samples prepared from commercially available pooled plasma (from Seralabs) were assayed with every 20 study participant samples. The overall coefficient of variation was 4.3% at a mean concentration of 10.8 nmol/L. The lower limit of detection was 1.3 nmol/L for the IDS‐iSYS immunoassay, adequate to detect the lowest concentration in all study samples.

### Statistical analysis

Differences in baseline characteristics of cases and controls were compared using paired *t*‐tests for continuous variables and *χ* ^2^ tests or conditional logistic regression models for categorical variables, comparing the value for the case with the value in the matched control participant. For cases only, years between blood collection and diagnosis, age at diagnosis and type of lymphoid malignancy were additionally reported.

For all analyses, concentrations of IGF‐I were transformed logarithmically to approximate a normal distribution. Conditional logistic regression models were applied to calculate the relative risks (odds ratios [ORs]) for total lymphoma, BCL subtypes, T‐cell lymphoma, and HL in relation to thirds of IGF‐I concentration using cut points defined by the sex‐specific tertiles among control participants for all centres combined and using the lowest category as reference. Analyses of risk of overall lymphoma were conditioned on the matching variables, and were also conducted with additional adjustment for smoking (never, past, current), body mass index (BMI, kg/m^2^; in fourths), physical activity (inactive, moderately inactive, moderately active/active),^15^ alcohol intake (<8, 8–15, 16–39, ≥40 g/day), marital status (married/cohabiting or not married/cohabiting) and education level (primary or equivalent, secondary, degree level). For each of these variables a small proportion of values were unknown (participants with missing data on the covariates were assigned to an “unknown” category; <11% of values missing for each, with the exception of marital status, for which 32% of values were missing); these observations were included in the analyses as a separate “unknown” category. For subtypes of BCL, and for T‐cell and HL, analyses were conditioned on the matching variables but were not conducted with additional adjustments because of the small number of cases for these less common lymphoma subtypes. Tests for linear trend were obtained using a continuous variable with values equal to the median concentration within each tertile of plasma IGF‐I concentration.

The heterogeneity of the ORs by sex, age at blood collection (<60 or ≥60 years), time between blood collection and diagnosis (<48 or ≥48 months), age at diagnosis (<60 or ≥60 years) and BMI (<25 or ≥25 kg/m^2^), was examined using likelihood ratio *χ* ^2^ tests, based on models with and without an interaction term between IGF‐I concentration and the variable of interest.

Statistical analyses were performed with the Stata 14.0 statistical software package.^16^ All tests of statistical significance were two‐sided and *p* values below 0.05 were considered significant.

## Results

The baseline characteristics of the 1,072 lymphoma cases and 1,072 participants without lymphoma (controls) are provided in Table 1. Case and control subjects were similar with respect to age, BMI, smoking status, physical activity, marital status, and educational level. The overall median age at blood collection was 57.5 years (range: 29.6–79.2 years). Participants with lymphoma were diagnosed an average 4.9 years after blood collection (range: <1–13 years) and the median age at diagnosis was 61.9 years (range: 33–83 years).

**Table 1:** Characteristics of lymphoma cases and matched controls participants in EPIC

	**Cases (n=1072)**	**Controls (n=1072)**	***p*** a
Male, *n* (%)	559 (52.1)	559 (52.1)	
Age at blood collection, yr^b^	57.5 (8.0)	57.5 (8.0)	
Age at diagnosis, yr^b^	61.9 (8.1)		
Years between blood collection and diagnosis b	4.9 (2.8)		
BMI (kg/m^2^)^b^	26.4 (4.2)	26.3 (3.9)	0.5
Alcohol at recruitment (g/d)^b^	1.94 (1.16)	1.92 (1.17)	0.7
Smoking status, *n* (%)			0.4
Never	428 (39.9)	456 (42.5)	
Former	382 (35.6)	347 (32.4)	
Current smoker	247 (23.0)	257 (24.0)	
Unknown	15 (1.4)	12 (1.1)	
Physical activity, *n* (%)			0.9
Inactive	237 (22.1)	237 (22.1)	
Moderately inactive	307 (28.6)	306 (28.5)	
Moderately active	201 (18.8)	206 (19.2)	
Active	209 (19.5)	212 (19.8)	
Unknown	118 (11.0)	111 (10.4)	
Marital status, *n* (%)			0.7
Married	580 (54.1)	577 (53.8)	
Single	136 (12.7)	148 (13.8)	
Unknown	356 (33.2)	347 (32.4)	
Education, *n* (%)			0.3
Primary school/none	396 (36.9)	436 (40.7)	
Secondary	439 (41.0)	411 (38.3)	
Degree	193 (18.0)	187 (17.4)	
Unknown	44 (4.1)	38 (3.5)	
Lymphoma type, *n* (%)			
BCL	897 (83.7)		
DLBCL	124 (11.6)		
FL	115 (10.7)		
B‐CLL	184 (17.2)		
MM	237 (22.1)		
Other subtypes of BCL^c^	237 (22.1)		
T‐NHL	34 (3.2)		
HL	51 (4.8)		
Other subtypes of lymphoma	90 (8.4)		
IGF‐I (nmol/L)^d^	15.5 (15.3–15.8)	15.9 (15.6–16.2)	0.05

a Two‐sided p values for difference from paired t‐test, comparing concentrations within matched case control pair and χ2 test for categorical variables.

b Mean (SD).

c Those cases for which the BCL subtype is unknown or does not fall within the more common BCL subtypes (*i.e*. DBCL, FL, B‐CLL or MM).

d Geometric mean (95% CI).

Abbreviations: *N*, number; BCL, B‐cell lymphoma; B‐CLL, B‐cell chronic lymphocytic leukemia; DLBCL, diffuse large B‐cell lymphoma; FL, follicular lymphoma; HL, Hodgkin lymphoma; IGF‐I, insulin‐like growth factor I; MM, multiple myeloma; NHL, non‐Hodgkin lymphoma.

Age at blood collection, BMI, and geometric mean circulating concentrations of plasma IGF‐I of case patients and control participants are shown in Supporting Information Table 1. Mean IGF‐I concentration was slightly lower in cases than controls (15.5 *vs*. 15.9 nmol/L, respectively; *p =* 0.05), and this difference was particularly evident for those diagnosed with “other subtypes of BCL” (unknown BCL subtype or does not fall within the more common BCL subtypes) (15.2 *vs*. 15.9 nmol/L in cases *vs*. controls, *p =* 0.004).

The association between circulating IGF‐I concentration and lymphoma risk is shown in Table 2. After adjustment for smoking, physical activity, alcohol intake, marital status, education and BMI, the OR for the highest versus the lowest third of IGF‐I concentration was 0.82, 95% CI: 0.65–1.02; *p*_trend_ = 0.06 for overall lymphoma. Analyses of the association of IGF‐I concentration with rarer subtypes of lymphoma showed no significant association with DLBCL, FL, B‐CLL, MM, T‐NHL or HL, although numbers of cases were small.

**Table 2:** Odds ratios (95% confidence intervals) for all lymphoma and for lymphoma subclasses by third of IGF‐I concentration

	Third of IGF‐I			
	1 (Reference)	2	3	*p* for trend^a^
All lymphoma				
Cases/controls, *n*	397/358	339/357	336/357	
OR (95% CI)^b^	1.00 (ref)	0.85 (0.69–1.05)	0.83 (0.67–1.04)	0.05
Multivariable‐adjusted OR (95% CI)^c^	1.00 (ref)	0.85 (0.69–1.05)	0.82 (0.65–1.02)	0.06
BCL subtypes^d^				
DLBCL				
Cases/controls (*n*)	33/40	50/46	41/38	
OR (95% CI)^b^	1.00	1.31 (0.72–2.36)	1.34 (0.68–2.64)	0.6
FL				
Cases/controls (*n*)	44/38	35/32	36/45	
OR (95% CI)^b^	1.00	0.94 (0.50–1.77)	0.66 (0.34–1.27)	0.2
B‐CLL				
Cases/controls (*n*)	67/56	56/66	61/62	
OR (95% CI)^b^	1.00	0.71 (0.42–1.17)	0.82 (0.48–1.39)	0.3
MM				
Cases/controls (*n*)	99/92	68/80	70/65	
OR (95% CI)^b^	1.00	0.81 (0.54–1.22)	1.00 (0.64–1.56)	0.6
Other subtypes of BCL^e^				
Cases/controls (*n*)	97/75	72/63	68/99	
OR (95% CI)^b^	1.00	0.90 (0.57–1.41)	0.46 (0.28–0.75)	0.004
T‐NHL				
Cases/controls (*n*)	11/12	12/13	11/9	
OR (95% CI)^b^	1.00	1.08 (0.25–4.70)	1.40 (0.34–5.81)	0.6
HL				
Cases/controls (*n*	13/15	18/20	20/16	
OR (95% CI)^b^	1.00	1.15 (0.41–3.18)	1.85 (0.52–6.60)	0.5
Other subtypes of lymphoma				
Cases/controls (*n*)	33/30	28/37	29/23	
OR (95% CI)^b^	1.00	0.68 (0.30–1.52)	1.11 (0.45–2.75)	0.5

Case patients and control participants were matched on recruitment centre, age at enrolment (±6 months), time of day of blood collection (±1 hr), follow‐up time (as close as possible), time between blood draw and last consumption of food or drinks (<3, 3–6, >6 hr).

a *p* Value from test of trend on 1 *df* based on continuous log concentration.

b ORs (95% CIs) are from conditional logistic regression models conditioned on the matching variables (above) but without additional adjustments due to the small number of cases for these lymphoma subtypes.

c ORs (95% CIs) are from conditional logistic regression models conditioned on the matching variables (above) and additionally adjusted for smoking (never, past, present), physical activity (inactive, moderately inactive, moderately active and active), alcohol intake (<8 g/day, 8–15 g/d, 16–39 g/d, ≥40 g/d), marital status (married or cohabiting, not married or cohabiting), education (primary or none, secondary, degree level) and BMI (sex‐specific quartiles).

d BCL includes DLBCL, FL, B‐CLL, MM and other subtypes of BCL.

e Those cases for which the BCL subtype is unknown or does not fall within the more common BCL subtypes (*i.e*. DBCL, FL, B‐CLL or MM).

Abbreviations: *N*, number; BCL, B‐cell lymphoma; B‐CLL, B‐cell chronic lymphocytic leukemia; DLBCL, diffuse large B‐cell lymphoma; FL, follicular lymphoma; HL, Hodgkin lymphoma; IGF‐I, insulin‐like growth factor I; MM, multiple myeloma; NHL, non‐Hodgkin lymphoma.

Finally, there was no evidence of heterogeneity in the association of IGF‐I and risk of overall lymphoma (Table 3) by sex (*p*_heterogeneity_ = 0.08), age at blood collection (*p*_heterogeneity_ = 0.3), time between blood collection and diagnosis (*p*_heterogeneity_ = 0.9), age at diagnosis (*p*_heterogeneity =_ 0.2), or BMI (*p*_heterogeneity_ = 0.09).

**Table 3:** Odds ratios (95% confidence intervals) for all lymphoma by third of IGF‐I concentration, subdivided by selected factors

	Third of IGF‐I				
	1 (ref.)	2	3	*p* for trend^a^	*p* for het. of trends
Overall					0.06
Cases/controls (*n*)	397/358	339/357	336/357		
OR (95% CI)^b^	1.00	0.85 (0.69–1.05)	0.82 (0.65–1.03)	0.06	
Sex					0.08
Men					
Cases/controls (*n*)	187/187	182/186	190/186		
OR (95% CI)^b^	1.00	0.98 (0.73–1.32)	1.02 (0.74–1.40)	0.8	
Women					
Cases/controls (*n*)	210/171	157/171	146/171		
OR (95% CI)^b^	1.00	0.73 (0.53–1.01)	0.65 (0.46–0.90)	0.003	
Age at blood collection					0.3
<60 yr					
Cases/controls (*n*)	198/165	194/207	231/251		
OR (95% CI)^b^	1.00	0.76 (0.57–1.01)	0.73 (0.54–1.01)	0.02	
≥60 yr					
Cases/controls (*n*)	192/183	139/145	99/102		
OR (95% CI)^b^	1.00	0.91 (0.66–1.25)	0.89 (0.60–1.31)	0.6	
Time between blood collection and diagnosis					0.9
<48 mo					
Cases/controls (*n*)	151/157	147/130	135/146		
OR (95% CI)^b^	1.00	1.20 (0.84–1.70)	0.96 (0.66–1.41)	0.3	
≥48 mo					
Cases/controls (*n*)	246/201	192/227	201/211		
OR (95% CI)^b^	1.00	0.71 (0.54–0.93)	0.74 (0.55–0.99)	0.1	
Age at diagnosis^c^					0.2
<60 yr					
Cases/controls (*n*)	92/90	118/133	175/162		
OR (95% CI)^b^	1.00	0.84 (0.56–1.25)	1.02 (0.68–1.53)	0.9	
≥60 yr					
Cases/controls (*n*)	305/268	221/224	161/195		
OR (95% CI)^b^	1.00	0.88 (0.68–1.14)	0.68 (0.51–0.91)	0.03	
Body mass index					0.09
<25 kg/m^2^					
Cases/controls (*n*)	72/53	61/73	61/68		
OR (95% CI)^b^	1.00	0.64 (0.38–1.08)	0.59 (0.33–1.06)	0.01	
≥25 kg/m^2^					
Cases/controls (*n*)	166/142	119/141	128/126		
OR (95% CI)^b^	1.00	0.70 (0.50–1.00)	0.76 (0.52–1.12)	0.3	

Case patients and control participants were matched on recruitment centre, age at enrolment (±6 months), time of day of blood collection (±1 hr), follow‐up time (a0073 close as possible), time between blood draw and last consumption of food or drinks (<3, 3–6, >6 hr).

a *p* Value from test of trend on 1 *df* based on continuous log concentration.

b ORs (95% CIs) are from conditional logistic regression models conditioned on the matching variables (above) and additionally adjusted for smoking (never, past, present), physical activity (inactive, moderately inactive, moderately active and active), alcohol intake (<8 g/d, 8–15 g/d, 16–39 g/d, ≥40 g/d), marital status (married or cohabiting, not married or cohabiting), education (primary or none, secondary, degree level) and BMI (sex‐specific quartiles).

c Age at diagnosis for cases, and for each control their corresponding age at the date of diagnosis of the matched case.

Abbreviations: *N*, number; IGF‐I, insulin‐like growth factor I.

## Discussion

In this large European prospective study, plasma concentration of IGF‐I was not associated with overall risk for lymphoma.

To date, most of the data on the relationship between IGF‐I with risk of overall lymphoma come from *in vitro* or *in vivo* studies, and to our knowledge, this is the first large‐scale prospective epidemiological study to examine this association. One previous prospective study assessed the association between pre‐diagnosis IGF‐I and risk of MM and found no significant association.^11^ In the few clinical and retrospective studies of the IGF axis in relation to lymphoma, circulating IGF‐I concentrations have been found variously to be low in 84 survivors of childhood NHL,^17^ to not differ at diagnosis between 57 MM patients and 20 controls,^9^ and to be related to prognosis for MM patients (with low serum IGF‐I at diagnosis being associated with favourable prognosis).^18^ A recent study in classical HL patients has shown that despite the oncogenic effect of IGF‐IR, a higher expression at diagnosis predicted a favourable outcome both for overall survival and 5‐year progression‐free survival.^19^ In the current prospective study, the association between IGF‐I and overall lymphoma risk was close to significant; however, we had relatively limited sample size and lymphoma is a heterogeneous disease.^20^ Therefore, further prospective analyses of the association in studies with information on lymphoma subtype are warranted.

We observed that IGF‐I concentrations were not associated with the incidence of the common BCL subtypes (DLBCL, FL, MM, B‐CLL and MM), although the number of cases for most subtypes was small. We found an inverse association between IGF‐I and “other subtypes of BCL”, although this category includes those cases for which the BCL subtype is unknown or does not fall within the more common BCL subtypes (*i.e*. DBCL, FL, B‐CLL or MM), and therefore conclusions on risk cannot be drawn.

While there was no significant heterogeneity by time between blood collection and diagnosis, there was a suggestion of association only in those diagnosed 2 or more years after recruitment and not in those diagnosed in the first 2 years of follow‐up. Further prospective data are needed, but these results suggest that reverse causality is unlikely to account for our findings. Also, we found no evidence of significant heterogeneity by sex, age at blood collection, age at diagnosis, or body mass index. However, significant trends in women, in person 60 years and older, and in normal weight participants were found. It is possible that we did not observe heterogeneity by sex, age and BMI because of the limited sample size in subgroups and, therefore, further prospective data are needed to explore possible potential differences by sex, age and BMI.

Strengths of the current analysis include the sample size, the length of follow‐up and the information available on lymphoma subtypes. The distribution of IGF‐I values among controls in this study is similar to that observed in previous studies.^21^

This study also has some limitations. IGF‐I concentrations were measured only once; however, previous studies reported moderately to high within‐individual reproducibility for IGF‐I measured in repeated blood samples collected up to 5 years apart (intraclass or Spearman rank correlations ranging between 0.4 and 0.9).^22^, ^23^, ^24^, ^25^ There was also a small number of participants' in some of the lymphomas subclasses and subgroups defined by participants characteristics, and also a relatively high proportion of BCL not assigned to a specific BCL‐subtype, limiting the statistical power to detect an association with specific lymphoma subtypes and subgroups. We were not able to investigate the role of early life factors, such as prenatal growth. It remains possible that under‐ and overnutrition *in utero* may lead to an elevated risk of developing lymphoma in later life and to reprogramming of the IGF‐I axis. Previous studies have found heterogeneity by sample media (*e.g*. serum or plasma) or type of assay,^2^ and this should be taken into consideration in future studies. It is also not possible to rule out residual confounding; in particular we were unable to investigate potential confounding factors related to non‐steroidal anti‐inflammatory drugs, infections or birthweight. Finally, data on other IGFs and IGF‐binding proteins were not available in the current study.

In conclusion, these results suggest no association between IGF‐I and overall lymphoma risk. Further prospective data are required to investigate the relationship of IGF‐I with lymphoma subtypes.

## Supporting information

Supporting Information Figure 1

Supporting Information Table 1
