# Supplementary material for: Prediagnostic circulating concentrations of plasma insulin‐like growth factor‐I and risk of lymphoma in the European Prospective Investigation into Cancer and Nutrition
Source: Int J Cancer. 2016 Dec 27;140(5):1111–8. doi: 10.1002/ijc.30528 (PMC5299544; doi:10.1002/ijc.30528)
Supplement: Supplementary file 1 — Supporting Information Figure 1 [file IJC-140-1111-s001.docx]

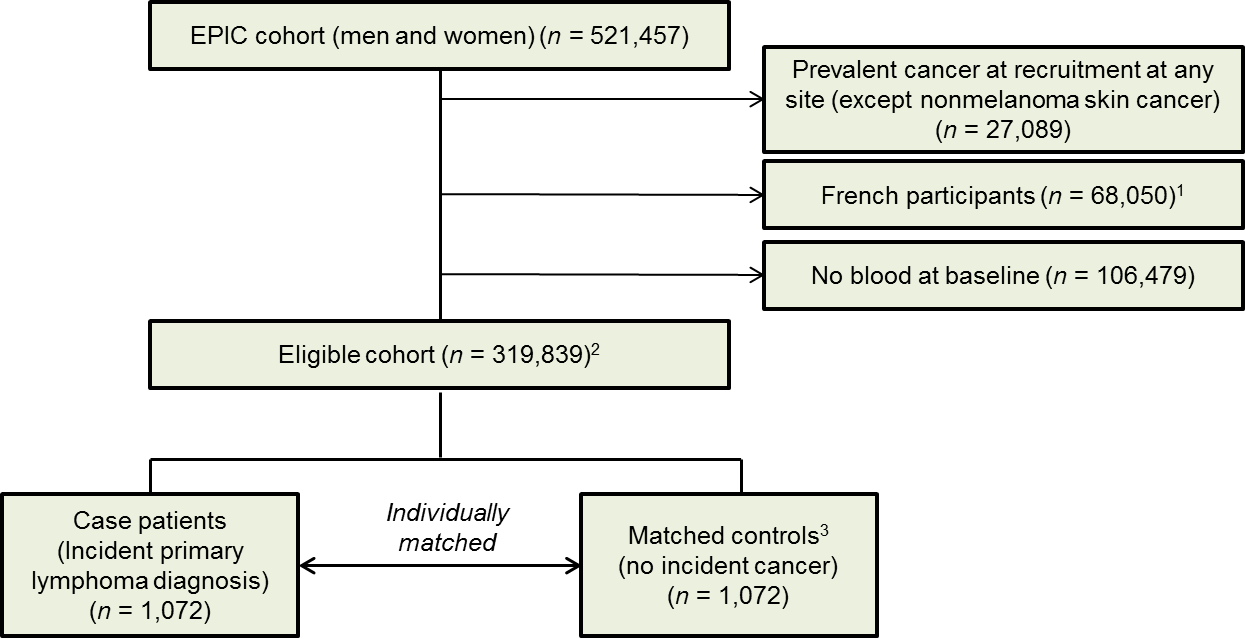


**Supplemental Figure 1**. Flow diagram of participant selection.

^1^ Participants in the French cohort were excluded from the study because of incomplete coding for lymphoid neoplasms.

^2^ Donated blood sample at baseline.

^3^ Control patients were matched on recruitment centre, age at enrolment (± 6 months), time of day of blood collection (± 1 hour), follow-up time (as close as possible), time between blood draw and last consumption of food or drinks (<3, 3-6, >6 hours) using an incidence density sampling procedure.
