# Supplementary material for: Prediagnostic circulating concentrations of plasma insulin‐like growth factor‐I and risk of lymphoma in the European Prospective Investigation into Cancer and Nutrition
Source: Int J Cancer. 2016 Dec 27;140(5):1111–8. doi: 10.1002/ijc.30528 (PMC5299544; doi:10.1002/ijc.30528)
Supplement: Supplementary file 2 — Supporting Information Table 1 [file IJC-140-1111-s002.docx]

**Supplemental table 1.** Age at recruitment, body mass index and geometric mean circulating concentrations of plasma IGF-I of participants, stratified by case control status and by sub-classes of lymphoma.

|  | **Number** | **Age (SD)** | **BMI (SD)** | **Geometric mean IGF-I (95% CI)** | | ***P*-value^a^** |
| --- | --- | --- | --- | --- | --- | --- |
| **All participants** | 2144 | 57.5 (8.0) | 26.4 (4.1) | 15.7 | (15.5-15.9) | - |
| All controls | 1072 | 57.5 (8.0) | 26.3 (3.9) | 15.9 | (15.6-16.2) | - |
| All lymphoma | 1072 | 57.5 (8.0) | 26.4 (4.2) | 15.5 | (15.3-15.8) | 0.05 |
| BCL^b^ |  |  |  |  |  |  |
| DLBCL | 124 | 55.9 (7.8) | 27.1 (4.5) | 16.1 | (15.3-16.9) | 0.6 |
| FL | 115 | 55.8 (7.5) | 25.5 (3.8) | 15.4 | (14.6-16.4) | 0.2 |
| B-CLL | 184 | 57.9 (7.5) | 26.8 (4.6) | 15.7 | (15.0-16.4) | 0.3 |
| MM | 237 | 58.1 (7.7) | 26.7 (4.3) | 15.1 | (14.9-15.8) | 0.6 |
| Other subtypes of BCL^c^ | 237 | 59.1 (7.6) | 26.1 (4.1) | 15.2 | (14.5-15.7) | 0.004 |
| T-NHL | 34 | 58.6 (7.8) | 27.4 (4.0) | 16.2 | (14.5-18.2) | 0.6 |
| HL | 51 | 52.3 (9.6) | 26.4 (4.0) | 16.5 | (15.4-17.8) | 0.6 |
| Other subtypes of lymphoma | 90 | 57.4 (8.9) | 25.8 (3.6) | 15.9 | (14.8-17.1) | 0.5 |

BCL, B-cell lymphoma; B-CLL, B-cell chronic lymphocytic leukemia; DLBCL, diffuse large B-cell lymphoma; FL, follicular lymphoma; HL, Hodgkin lymphoma; IGF-I, insulin-like growth factor I; MM, multiple myeloma; NHL, non-Hodgkin lymphoma.

Case patients and control participants were matched on recruitment centre, age at enrolment (± 6 months), time of day of blood collection (± 1 hour), follow-up time (as close as possible), time between blood draw and last consumption of food or drinks (<3, 3-6, >6 hours).

^a^Two-sided P-values for difference from paired t-test, comparing IGF-I concentrations within matched case control pair, i.e. between cases of that lymphoma sub-class and their matched control.

^b^BCL includes DLBCL, FL, B-CLL, MM and other subtypes of BCL.

^c^Those cases for which the BCL subtype is unknown or does not fall within the more common BCL subtypes (i.e. DBCL, FL, B-CLL or MM).
